# Diagnosis of Follicular Lesions of Undetermined Significance in Fine-Needle Aspirations of Thyroid Nodules

**DOI:** 10.1155/2013/250347

**Published:** 2013-03-24

**Authors:** J. Ratour, M. Polivka, H. Dahan, L. Hamzi, R. Kania, M. L. Dumuis, R. Cohen, M. Laloi-Michelin, B. Cochand-Priollet

**Affiliations:** ^1^Department of Pathology, Lariboisière Hospital, Assistance Publique-Hôpitaux de Paris, Paris 7 University, Paris Sorbonne Cité, 75475 Paris Cedex 10, France; ^2^Department of Radiology, Lariboisière Hospital, Assistance Publique-Hôpitaux de Paris, Paris 7 University, Paris Sorbonne Cité, 75475 Paris Cedex 10, France; ^3^Departments of Laryngology, Otology, and Cervico-Facial Surgery, Lariboisière Hospital, Assistance Publique-Hôpitaux de Paris, Paris 7 University, Paris Sorbonne Cité, 75475 Paris Cedex 10, France; ^4^Department of Endocrinology, Avicenne Hospital, Assistance Publique-Hôpitaux de Paris, Paris 13 University, Paris Sorbonne Cité, 93000 Bobigny, France; ^5^Department of Endocrinology, Lariboisière Hospital, Assistance Publique-Hôpitaux de Paris, Paris 7 University, Paris Sorbonne Cité, 93000 Bobigny, France

## Abstract

*Aim*. We aimed to analyze the diagnostic criteria proposed by the Bethesda System for Reporting Thyroid Cytopathology for follicular lesions of undetermined significance (FLUS), the risk of cancer and diagnostic improvement with use of immunocytochemistry. *Methods*. For each FLUS diagnosis, we analyzed the cytological criteria (9 Bethesda criteria), secondary fine-needle aspiration (FNA) results, surgical procedures, contribution of immunocytochemistry with the antibodies cytokeratin 19 (CK19) and monoclonal anti-human mesothelial cell (HBME1). *Results*. Among patients with 2,210 thyroid FNAs, 244 lesions (337 nodules) were classified as FLUS (11% of all thyroid FNAs). The 3 criteria most often applied were cytological atypia suggesting papillary carcinoma (36%), microfollicular architecture but sparse cellularity (23.1%), cytological atypia (21.5%). With secondary FNA, 48.8% of nodules were reclassified as benign. For about half of all cases (41.4% for the first FNA, 57.6% for the second FNA), immunocytochemistry helped establishing a diagnosis favoring malignant or benign. No benign immunocytochemistry results were associated with a malignant lesion. In all, 22.5% of the 39 removed nodules were malignant. *Conclusion*. The FLUS category is supported by well-described criteria. The risk of malignancy in our series was 22.5%. Because we had no false-negative immunocytochemistry results, immunocytochemistry could be helpful in FLUS management.

## 1. Introduction

Thyroid nodules are common in the general population, with a clinical prevalence between 5.3% and 6.4% for women and between 0.8% and 1.6% for men in normally “iodine countries” [1]. The prevalence seen on ultrasonography is 10 times higher between 11% and 50% [1]. Only 5% of these nodules are malignant [1]. 

Thyroid fine-needle aspiration (FNA) represents a simple, fast, reliable, and minimally invasive technique to explore nodules. Only malignant lesions should be excised. Considering its high sensitivity—usually more than 90%—thyroid FNA was recommended by international experts as a preliminary or screening test to detect thyroid cancer [2–5]. Nevertheless, thyroid FNA has low specificity—usually 50% to 65%—which leads to many unnecessary surgical procedures. Only 20% to 30% of all indeterminate cases by FNA were found malignant on histology, with high variability in assessing risk of malignancy depending on the terminology used [6, 7].

The last several years have seen the publication of international classifications for thyroid lesions based on FNA results, more or less linked with ultrasonography data. In 2006, the American Thyroid Association and the Italian Society of Pathology and Cytology [8] published a 4-tiered classification: nondiagnostic, benign, malignant, and suspicious or indeterminate. The 2009 American Thyroid Association classification [9] was nondiagnostic, malignant, indeterminate or suspicious for neoplasm, and benign. In 2010, the British Thyroid Association proposed a 5- to 8-tiered terminology, and the US National Cancer Institute [10] published the Bethesda System For Reporting Thyroid Cytopathology (BSRTC) with 6 categories: benign; follicular lesion of undetermined significance/atypia of undetermined significance (FLUS/AUS); follicular neoplasm/follicular neoplasm Hürthle-cell variant; suspicious for malignancy; malignant; and nondiagnostic (unsatisfactory). The BSRTC is the only system that gives a detailed description of the diagnostic criteria (“the Bluebook”) [11], the expected risk of malignancy for each category, and recommendations for management.

The FLUS/AUS category is heterogeneous in including cases for which the cytological findings are not convincingly benign. It is associated with an estimated risk of malignancy between 5% and 15%, so only a few patients should undergo surgery. This category was recommended to represent no more than 7% of thyroid FNA reports. The first published studies suggested great variability in the application of this category, with percentages ranging from 2.5% to 28.6% of the FNA [12–24].

We aimed to use our series of thyroid abnormalities to analyze the cytological criteria of the FLUS diagnosis, secondary FNA results, recommendations for surgery when available, and the contribution of immunocytochemistry with 2 antibodies to improve the diagnosis, as recently reported [25].

## 2. Materials and Methods

In all, 2,210 thyroid FNAs were performed during a 2-year period, 2010 to 2011, in our institutions. Radiologists from 2 centers, Avicenne Hospital and Lariboisière Hospital, both belonging to the Assistance Publique-Hôpitaux de Paris institution in France, performed the thyroid FNAs under ultrasonography guidance using 23- to 25-gauge needles. For all these cases, the cytological material was analyzed on liquid-based slides (Thinprep Hologic). 1 superfrost slide was prepared following the usual guidelines of the firm (Hologic) and analyzed after Papanicolaou staining.

Tissues from all abnormal cases, including FLUS, to malignant cases but not cases with insufficient cellularity were examined by immunocytochemistry. Two antibodies, cytokeratin 19 (CK19; Novocastra 1/100) and HBME1 (Dako 1/50), were systematically used together. 2 or 3 additional liquid-based slides were prepared. An automated technique was used (Ventana BMK). Detection was done by the use of the avidin biotin complex with DAB chromogen, without epitope retrieval.

Immunocytochemistry results were classified by 4 categories: noncontributive (<5 remaining sheets of follicular cells), favoring benign (<30% positive cells for both antibodies), favoring malignant (>70% positive cells for both antibodies), and indeterminate (30–70% positive cells for both antibodies and results for both antibodies not falling into the previous categories). The report stated FLUS and the ICC status was added.

The BSRTC includes 9 well-described criteria for FLUS: (i) microfollicular architecture, but sparse cellularity, (ii) predominant oncocytic cells and low cellularity, (iii) predominant oncocytic cells and goiter or Hashimoto thyroiditis, (iv) cytological atypia suggesting papillary carcinoma, (v) cytological atypia, (vi) cytological atypia due to technical artifact, (vii) atypical “cyst lining cells,” (viii) abnormal lymphocytic population, and (ix) other. We noted all cytological findings described by pathologists. 

All thyroid FNAs with an FLUS diagnosis were selected by computerized search. All cytological reports were studied by patient characteristics, cytological criteria leading to an FLUS diagnosis, immunocytochemistry results, and results of secondary FNAs and histological controls, when available. Data were analyzed by the use of Microsoft Excel.

## 3. Results

For the BSRTC categories, from the 2,210 thyroid FNAs, 1,434 FNAs were benign (65.5%), 244 were FLUS/AUS (11%), 109 were follicular neoplasms (4.9%), 52 were suspicious for malignancy (2.3%), 46 were malignant (2%) and 309 were non-diagnostic (14.3%). FLUS concerned 182 women and 62 men, that is, 244 patients and 337 nodules (some patients with 2 or more nodules classified as FLUS at the first FNA). The largest numbers of patients were between 50 and 60 years old(mean age 53.2 years; Table 1). More than half of the patients presented goiter (58.1%) and 27.5% single nodules. The mean size of nodules was 23.4 mm (range 7 to 80 mm; Table 1). Overall, 86 secondary FNAs were performed (25.5%): most gave benign results (*n* = 42, 48.8%); 31.4% (*n* = 27) were reclassified in another category: suspicious for malignancy 13.9% (*n* = 12), follicular neoplasm was 7% (*n* = 6), and nondiagnostic was 10.5% (*n* = 9), and the remaining cases remained as FLUS (19.8%, *n* = 17). No malignant lesion was diagnosed on secondary FNA.

For cytological criteria (Tables 2 and 3), 511 criteria were collected from the 354 FLUS nodules (337 FLUS from the first FNA, 17 additional FLUS from the second FNA, and more than one criteria for several reports). The most frequently applied criteria were cytological atypia suggesting a papillary carcinoma (36%), microfollicular architecture but sparse cellularity, and cytological atypia, for 23.1% and 21.5%, respectively. The categories of predominant oncocytic cells with low cellularity and predominant oncocytic cells and goiter or Hashimoto thyroiditis were applied to only 3.7% and 0.6% of nodules, respectively. We found no cases of abnormal lymphoid population or cytological atypia due to artifacts or cyst lining cells. Cases in the “other” category included those with some atypical giant cells (2.5%), low cellularity with few atypia (11.7%), or lack of colloids (0.8%).

For the 337 nodules of the first FNA, 216 were analyzed by immunocytochemistry (64.1%) with both CK19 and HBME1 antibodies. The rates of benign, malignant, indeterminate, and noncontributory diagnoses were 38.5%, 2.9%, 46.5%, and 12.1% on immunocytochemistry (Table 4). For the second FNA, immunocytochemistry could be performed for 54.6% of these cases (*n* = 47), with the same 2 antibodies as for the first FNA (i.e., CK19 and HBME1). The results were favoring benign (44.9%), indeterminate (38.2%), favoring malignant (12.7%), and noncontributory (4.2%) (Table 4). Histology outcome data were available for 11.6% (39/337) of the FLUS nodules: 9 carcinoma (23%), including 6 papillary carcinoma, 1 papillary carcinoma follicular variant, 1 follicular carcinoma, and 1 metastasis of a pancreatic carcinoma. We found no false-negative immunocytochemistry results but found one false-positive result with the category favoring malignant.

## 4. Discussion

FLUS belongs to the “gray-zone” or “indeterminate” thyroid FNA results. It was proposed by the BSRTC as a specific category representing low risk of malignancy between 5% and 15%. Cases considered as FLUS are those for which cytological findings are not convincingly benign, but the degree of architectural and cellular atypia is also not sufficient for a diagnosis of follicular neoplasm or suspicious for malignancy.

In our series, the FLUS category represented 11% of all thyroid FNAs over a 2-year period (2010-2011). This represents a higher percentage than the recommended Bethesda rate (7%). Nevertheless, this percentage is one of the lowest in the literature (2.1% to 28.6%) [26, 27], since most of the published series have exhibited a rate higher than 10% (Table 3). More recent studies seem to report a stable rate around 12%, which reflects the need for training [28, 29]. In our study, the main criteria leading to the diagnosis of FLUS were represented by the categories of cellular atypia suggesting papillary carcinoma (36%), followed by microfollicular architecture but sparse cellularity and cytological atypia. 

Comparing our results with that from other series is difficult because the criteria leading to FLUS have usually not been described in other series. Some studies showed a relatively constant FLUS rate in the laboratory but notable variability between individual cytopathologists (6.1–18.7%) [27]. Thyroid experts are concerned by this poor interobserver reproducibility because consensus was achieved in only 62.1% of the cases in a recent study involving 4 experts; disagreement mainly occurred for the FLUS category (consensus achieved in 20% of cases) [12]. Thus, better training in the described cytological criteria leading to an FLUS diagnosis may be necessary.

We anticipated that the subcategories cytological atypia suggesting a papillary carcinoma (36%) and microfollicular architecture, but sparse cellularity would represent most of the FLUS. Of note, other subcategories, such as predominant oncocytic cells and goiter or Hashimoto thyroiditis, atypical lymphoid population, and cytological atypia due to artifacts as well as cyst lining cells were only rarely mentioned. For the latter 2 subcategories, some technical aspects might explain the results. Liquid-based cytology, with well-preserved cells, reduces the artifacts and allows for easy visualization of so-called cyst-lining cells. We found no abnormal lymphocytic populations, perhaps because of the absence of hematology departments in our hospitals, which therefore represents a bias of recruitment. The very low rate of the subcategory predominant oncocytic cells and goiter or Hashimoto thyroiditis is more likely due to insufficient use of these criteria by our team, considering that the risk of oncocytic tumors and therefore cancer is the same in a goiter than a unique nodule. However, this finding should be confirmed by a large study including a histological control.

The FLUS criterion “other” is interesting because pathologists have the opportunity to describe some additional details of their diagnosis with this subcategory. Some studies have reported rates combining nondiagnostic and FLUS categories, ranging from 8.9% to 32% [26] and thus leading to secondary FNA. In our series, the diagnosis of FLUS was associated with low cellularity in more than 10% of the reports (11.75%). This less-than-optimal quality increases the incidence of the FLUS diagnosis but also the incidence of indeterminate and/or noncontributive immunocytochemistry results. The link between poor cellularity and FLUS should be further studied.

The BSRTC recommends secondary FNA 3–6 months after an FLUS diagnosis. In our series, on secondary FNA, 48.8% of FLUS diagnoses were reclassified as benign and 31.4% as another category. These results are in agreement with others [30]. Some studies suggested the use of core-needle biopsy after an FLUS or nondiagnostic diagnosis, which would improve the diagnostic performance, thus decreasing the number of diagnoses of FLUS (23.6% versus 39.8%) and nondiagnostic (12.5% versus 43.5%) [13]. Nevertheless, this technique implies several risks (hemorrhage) and limitations (accessibility of the nodule) and is not widely performed. 

The low number of secondary FNAs (25.5%) and histological controls (11.6%) in our study could be explained by our use of immunocytochemistry combining 2 antibodies (CK19 and HBME1). On immunocytochemistry, 38.5% and 44.9% of the diagnoses from the first and second FNAs, respectively, were reclassified as benign, and 2.9% and 12.7%, respectively, were reclassified as malignant. No malignant lesions were associated with a benign immunocytochemistry result. We found only one false-positive result. The usefulness of immunocytochemistry was previously reported by our team in a series of 150 cases comparing immunocytochemistry results to histological controls [25]. Immunocytochemistry was helpful for benign and malignant triage of FLUS, follicular neoplasm and suspicious for malignancy. We observed no false-negative results too. Concerning these lesions, the sensitivity, specificity, and negative and predictive values were 100%, 85.2%, 100%, and 87.2%, respectively. Since the performance of this study, we consider FLUS with benign immunocytochemistry results as benign nodules, and secondary FNA is not systematically required. Therefore, because immunocytochemistry implements informations and recommendations, it limits the number of secondary FNA.

The risk of malignancy was higher in our series than the BSRTC expected rate (22.5% versus 5–15%) and higher than the risk from a personal study for the 2-year period 2009 to 2011, which found a 17.2% risk of malignancy, before our immunocytochemistry validation study [29] but is not very different from other results [28, 31]. This high percentage is generally agreed to be due to selection bias because usually only clinically or ultrasonography suspicious nodules undergo a surgical procedure. Our series contains double bias because of the clinical/ultrasonography and immunocytochemistry selection of nodules.

## 5. Conclusions

The BSRTC FLUS category was suspected to become a kind of “waste-basket” category for diagnosis of thyroid abnormalities [32, 33]. Nevertheless, our series supports this diagnosis with well-described criteria, which should be applied and analyzed in larger studies. The quality of thyroid FNAs (adequate cellularity) may avoid some diagnoses of FLUS, and training might lead to better reproducibility. Atypia suggesting a papillary carcinoma seems to be a frequently applied subcategory. Secondary FNA following an FLUS diagnosis as recommended by the BSRTC seems to be useful and leads to a better classification with most of the benign results.

Because we have found no false-negative immunocytochemistry results neither in this series nor in a previously published larger one with 150 histological control concerning all abnormal categories of BSRTC, we consider that ICC favor benign can be now taken into account for patient management and followup. The systematic use of the technique, here with CK19 and HBME1 antibodies, will be helpful for the management of FLUS and avoiding secondary FNA. By selecting patients that should be amenable for surgery, immunocytochemistry fulfills the requirement of patient selection. These techniques lead to more conservative management of thyroid nodules classified as FLUS on FNA and can decrease the number of unnecessary procedures.

## Figures and Tables

**Table 1:** Characteristics of patients with follicular lesions of undetermined significance (FLUS) according to the Bethesda System for Reporting Thyroid Cytopathology (BSRTC).

	Number of patients/number of nodules (*n* = 244/337)
Age, years (%)	
>80	1 (0.4)
80–70	27 (11)
70–60	49 (20)
60–50	72 (29.6)
50–40	42 (17.3)
40–30	37 (15.1)
30–20	14 (5.8)
20–today	2 (0.8)
Thyroid condition	
Goiter	142 (58.1)
Single nodule	67 (27.5)
Hashimoto thyroiditis	4 (1.7)
Basedow thyroiditis	2 (0.8)
Other thyroiditis	17 (7.0)
Hyperthyroiditis	2 (0.8)
Hypothyroiditis	4 (1.7)
NS	6 (2.4)
Nodule side	
Left	155 (46)
Right	155 (46)
Isthmus	10 (2.9)
NS	17 (5.1)
Nodule size, mm	
<10	17 (5.1)
10–20	116 (34.4)
21–30	77 (22.9)
31–40	32 (9.5)
41–50	23 (6.8)
>50	10 (2.9)
NS	62 (18.4)

Data are number (%).

NS: not specified.

**Table 2:** Cytological criteria for FLUS diagnosis according to the BSRTC.

Microfollicular architecture but sparse cellularity
Predominant oncocytic cells and low cellularity
Predominant oncocytic cells and goiter or Hashimoto thyroiditis
Cytological atypia suggesting papillary carcinoma
Cytological atypia
Cytological atypia due to technical artifact
Atypical “cyst lining cells”
Abnormal lymphocytic population
Other

**Table 3:** Distribution of FLUS criteria in our series.

Cytological criteria (511 for 354 FLUS)	Number of lesions (%)
Microfollicular architecture but sparse cellularity	118 (23.1)
Predominant oncocytic cells with low cellularity	19 (3.7)
Predominant oncocytic cells and goiter or Hashimoto thyroiditis	3 (0.6)
Cytological atypia suggesting papillary carcinoma	184 (36.0)
Cytological atypia	110 (21.5)
Cytological atypia due to technical artifact	0 (0)
Atypical “cyst lining cells”	0 (0)
Abnormal lymphocytic population	0 (0)
Other	
Giant cells	13 (2.5)
Low cellularity	60 (11.7)
Low colloid	4 (0.8)

**Table 4:** Immunocytochemistry results for fine-needle aspirations (FNAs).

	First FNA	Second FNA
	*n* = 216	*n* = 47
Benign	38.5%	44.9%
Malignant	2.9%	12.7%
Indeterminate	46.5%	38.2%
Noncontributory	12.1%	4.2%
